# Investigating the effects of indoor lighting on measures of brain health in older adults: protocol for a cross-over randomized controlled trial

**DOI:** 10.1186/s12877-023-04594-7

**Published:** 2024-10-11

**Authors:** Kevin A. Mazurek, Linhao Li, Robert J. Klein, Shengliang Rong, Aidan F. Mullan, David T. Jones, Erik K. St. Louis, Gregory A. Worrell, Christina Y. Chen

**Affiliations:** 1 Well Living Lab, Rochester, MN USA; 2 Delos Living LLC, New York, NY USA; 3 Department of Physiology and Biomedical Engineering, Mayo Clinic, Rochester, MN USA; 4 Department of Neuroscience, University of Rochester, Rochester, NY USA; 5 Department of Quantitative Health Sciences, Mayo Clinic, Rochester, MN USA; 6 Department of Neurology, Mayo Clinic, Rochester, MN USA; 7 Department of Radiology, Mayo Clinic, Rochester, MN USA; 8 Center for Sleep Medicine, Mayo Clinic, Rochester, MN USA; 9 Department of Medicine, Mayo Clinic, Rochester, MN USA; 10 Department of Community Internal Medicine, Mayo Clinic, Rochester, MN USA

**Keywords:** Brain health, Cognitive function, Healthy aging, Indoor lighting, Physical activity, Sleep, Social engagement

## Abstract

**Background:**

The worldwide number of adults aged 60 years and older is expected to double from 1 billion in 2019 to 2.1 billion by 2050. As the population lives longer, the rising incidence of chronic diseases, cognitive disorders, and behavioral health issues threaten older adults’ health span. Exercising, getting sufficient sleep, and staying mentally and socially active can improve quality of life, increase independence, and potentially lower the risk for Alzheimer’s disease or other dementias. Nonpharmacological approaches might help promote such behaviors. Indoor lighting may impact sleep quality, physical activity, and cognitive function. Dynamically changing indoor lighting brightness and color throughout the day has positive effects on sleep, cognitive function, and physical activity of its occupants. The aim of this study is to investigate how different indoor lighting conditions affect such health measures to promote healthier aging.

**Methods:**

This protocol is a randomized, cross-over, single-site trial followed by an exploratory third intervention. Up to 70 older adults in independent living residences at a senior living facility will be recruited. During this 16-week study, participants will experience three lighting conditions. Two cohorts will first experience a static and a dynamic lighting condition in a cluster-randomized cross-over design. The static condition lighting will have fixed brightness and color to match lighting typically provided in the facility. For the dynamic condition, brightness and color will change throughout the day with increased brightness in the morning. After the cross-over, both cohorts will experience another dynamic lighting condition with increased morning brightness to determine if there is a saturation effect between light exposure and health-related measures. Light intake, sleep quality, and physical activity will be measured using wearable devices. Sleep, cognitive function, mood, and social engagement will be assessed using surveys and cognitive assessments.

**Discussion:**

We hypothesize participants will have better sleep quality and greater physical activity during the dynamic lighting compared to the static lighting condition. Additionally, we hypothesize there is a maximal threshold at which health-outcomes improve based on light exposure. Study findings may identify optimal indoor lighting solutions to promote healthy aging for older adults.

**Trial registration:**

ClinicalTrials.gov Identifier: NCT05978934.

## Background

According to the World Health Organization (WHO), the worldwide number of adults 60 years and older is expected to double from 1 billion in 2019 to 2.1 billion in 2050 [1]. As the population lives longer, the incidence of chronic diseases, cognitive disorders, and behavioral health changes will increase and negatively impact each adults’ health span. Although many age related health conditions are non-modifiable, certain lifestyle behaviors can help maintain or improve the brain and functional health of older adults as they age [2]. Regularly exercising and staying physically active has been shown to lower all-cause mortality in older adults [3, 4]. Sleep can also affect a multitude of health factors [5]. Poor sleep quality can impair cognitive performance [6] and increase the risk of dementia and all-cause mortality [7]. Socializing and staying mentally active are additional behaviors that can maintain or improve one’s brain health [8–11]. Adults who are socially isolated or lonely have been found to be at higher risk for heart disease, depression, and cognitive decline [12–16]. Thus, healthy lifestyle behaviors can increase older adults’ likelihood of remaining healthy later in life.

What can be done to promote such healthy lifestyle behaviors for older adults? One approach is to examine their day-to-day environment and determine whether modifying components of their living space may improve daily cognitive and physical stimulation. Indoor lighting could be a nonpharmacological approach to encourage healthy lifestyle behaviors that promote or maintain the brain health for older adults [17–19]. The importance of indoor lighting is often thought of in relation to seeing and performing activities. However, lighting has additional health-related benefits. For example, individuals who are exposed to more blue light during the daytime and less blue light during nighttime (e.g., dynamic lighting) often experience improvements in sleep quality [20–27]. This improvement in sleep quality could be due to increased suppression of melatonin secretion from the increased exposure to blue light in the morning, which helps entrain one’s circadian rhythm [28, 29]. Increasing the intensity of light in the morning also can improve the physical activity, cognitive function, and mood of adults exposed to the light [30–33]. Increasing the amount of ambient lighting in senior care facilities is also a preventative strategy to decrease fall risk [34]. However, there are saturation effects on subjective alertness and melatonin suppression when the light level reaches a certain threshold [35–39], and potentially negative effects such as increased agitation [40] or anxious/depressive behavior [41] if there is too much blue light exposure by people [42]. Thus, designing lighting systems specifically tailored for older adults’ residences is crucially important to promote their health and well-being and to improve their health spans.

Taking into consideration how older adults perceive light is crucial when designing indoor lighting systems to improve their health spans. Older adults need greater light intensities compared to younger adults to achieve health-related benefits [43–47]. Pupillary miosis and lens yellowing that occurs with age may explain the need for increased light intensities for older adults [48, 49]. Artificial light can be used to augment natural light as a therapy to improve sleep, mood, and general well-being [43]. Recent studies have shown that light therapy can significantly improve sleep disturbances, reduce agitation and depressive symptoms, and even enhance cognitive function in older individuals with dementia [50–55]. The efficacy of these natural light therapies may be due to activating intrinsically photosensitive retinal ganglion cells (ipRGCs), a subset of retinal ganglion cells in the eye that serve as the zeitgeber for the circadian system to regulate sleep-wake behavior [56–59]. However, activating ipRGCs outside of this pattern can increase the risk of developing circadian rhythm sleep-wake disorders, memory decline, and dementia [3, 43, 60, 61]. The timing, duration, brightness, and color of artificial light exposure have positive or negative effects on one’s physiological and psychological health [23, 35, 36, 62–75]. Understanding how the different lighting parameters positively or negatively affect the health of older adults is critical for improving their health span later in life.

This research protocol presents a novel approach to understanding how different indoor lighting conditions affect specific measures of brain health in older adults. Here, we are examining how three different lighting conditions affect measures of sleep quality, physical activity, cognitive performance, or social engagement by study participants. Our central hypothesis is each of these measures of brain health will increase when patients experience dynamic lighting conditions compared to static lighting conditions, because the health benefits of circadian lighting shown previously will subsequently improve sleep quality, cognitive performance, physical activity, and social engagement. To test our hypothesis, participants will each experience a dynamic and static lighting condition during which we will collect different measures related to their brain health. As a secondary hypothesis, we expect that the amount of blue light exposure saturates improvements in each measure of brain health, and we will examine this by comparing each measure of brain health between two different dynamic lighting conditions. Successful completion of this research protocol is expected to provide a better understanding about how measures of brain health of older adults could be improved by strategically designing the indoor lighting of their residences. Findings from this study could ultimately lead to optimal indoor lighting solutions and guidelines that maintain or improve the health and well-being of adults as they age.

## Methods/Design

### Ethical considerations

This study protocol involving human participants was reviewed and approved by The Mayo Clinic Institutional Review Board in March 2023, under the number 22-005400. Individuals will provide their written informed consent to participate in this study and this study. All experiments will be performed in accordance with relevant guidelines and regulations (such as the Declaration of Helsinki).

### Study aim

The purpose of this research study is to determine how indoor lighting affects different measures of brain and general health, such as sleep quality, physical activity, cognitive function, and social engagement, in older adults.

### Study setting

This study will be conducted in a senior living facility in Rochester, Minnesota, USA. Participants who live in the independent living residences, of which there are over 100 in the facility, will be recruited.

### Participants

Initially, up to 100 older adults will be screened into the study based on the inclusion exclusion criteria. However, because we are working with older adults where the incidence of cognitive impairment may be greater, we anticipate that up to 70 individuals will remain eligible after completing the consent capacity assessment (described below). Participants will be asked to allow study staff to install lighting in their residences that can be controlled remotely to produce the different lighting conditions. Up to 35 residences will be outfitted with the lighting systems resulting in between 35 and 70 participants being recruited (depending on whether the residence is single and double occupancy).

### Inclusion criteria

1. At least age 60;
2. Able to wear wearable devices throughout the study;
3. Willing to have their lighting in their kitchen and dining areas changed and controlled for the study;
4. Willing to have environmental sensors placed in their residence;
5. Willing to provide contact information about their primary care provider (PCP); and.
6. Able and has capacity to provide informed consent (score > 14.5 based on UBACC consent capacity form).

### Exclusion criteria

1. Is legally blind.
2. Previously renovated their living units and no longer have the standard lighting installation offered by the Senior Living Facility;
3. Currently spend or plan to spend most of their day outside of their residence during the study (i.e., would not experience the indoor lighting intervention for the majority of the study);
4. Plan to travel to a different time zone during the study; or.
5. Plan to be away from their residence for more than a week during the study.

Recruitment procedures will include word of mouth, flyers, and emails. There will also be presentations by members of the study team to potential study participants at the senior living facility to help answer questions or help with recruitment via word of mouth. Interested individuals can reach out to the study team to learn more and determine if they are eligible for the study. Any interested individual will be asked to complete an initial eligibility assessment.

### Initial eligibility assessment

During the Initial Eligibility Assessment, interested individuals will be provided a brief screening survey to determine if they meet the basic inclusion/exclusion criteria of the study. This will be provided electronically via Qualtrics. Participants who are eligible will then be provided the consent form to start the consenting process. Because the population we are recruiting is comprised of older individuals, there is some risk of severe cognitive impairment and/or decline among eligible survey respondents. Therefore, it is necessary to ensure each participant has the capacity to fully understand the study procedures and requirements before signing the consent form. A modified version of the University of California, San Diego Brief Assessment of Capacity to Consent (UBACC) will be administered to every individual after completing the digital consent form by a Mayo Clinic research coordinator. The UBACC has demonstrated high sensitivity and specificity and is an all-around strong assessment of one’s ability to understand the consent document before signing [76]. The UBACC will be administered and scored based on the guidelines from Jeste et al. Participants will need to score greater than 14.5 to be eligible for the study (as outlined in the inclusion criteria above).

We expect most participants to be deemed capable of consenting via the UBACC. However, we have buffered the total number of participants to account for individuals who do not meet inclusion criteria based on UBACC scores. Such participants will be provided an email explaining that (1) the nature of this study involves sophisticated components of technology solutions, monitoring, and data collection and (2) due to this, selection of participants needs to meet criteria to maximize the quality of intervention delivered while ensuring full understanding and safety for all participants involved.

Upon passing the UBACC, participants will be assigned the next available participant ID from a sequential pre-populated list. This ID will have been pre-assigned in inventory to all participant-specific item serial numbers (e.g., wearable sensors, light bulbs, etc.). Participants will then be initially randomly assigned to cohort based on a random number sequence and finally these cohorts will be stratified by gender. The Mayo Clinic research coordinator, who will not be involved in data analysis or collection, will be responsible for assigning IDs to cohorts. Participants will be blinded to which cohort and condition they are in.

### Baseline assessment/survey

A baseline survey will be used to collect information about each participant’s demographics, education level, current medications, and/or socioeconomic status.

### Study design: cross-over study design with an exploratory third condition

**Fig. 1:**
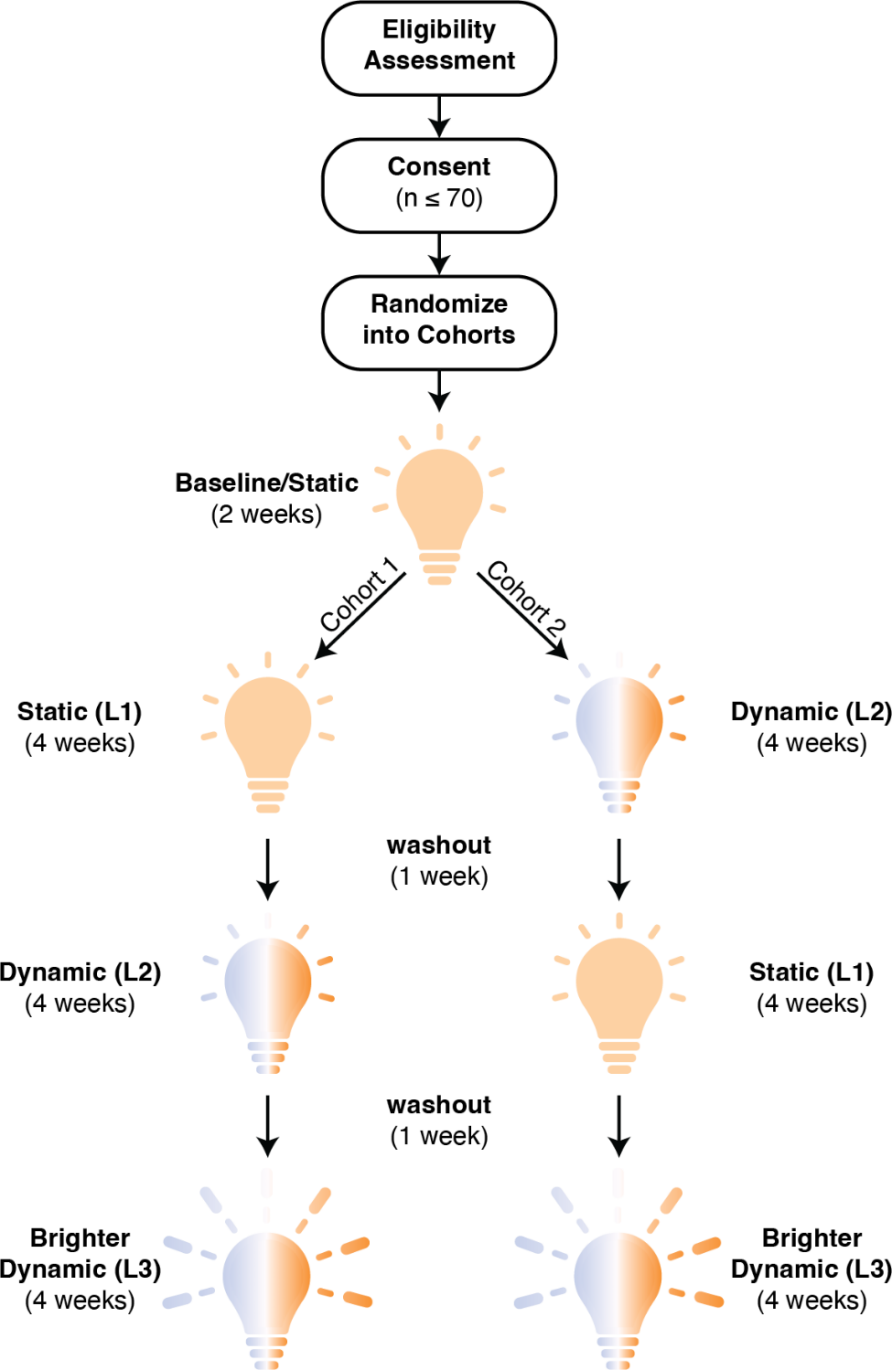
Schematic of the cross-over study design. Interested individuals will first perform an eligibility assessment to determine whether they can be consented into the study. Once 35 residences have been recruited (between 35 and 70 participants), participants will be randomized into two cohorts and experience three lighting conditions (L1, L2, L3). Lighting conditions are described in more detail in the “Environmental Lighting Conditions” section

The present study employs a two-arm, randomized, cross-over, single-site trial design. Participants will experience three 4-week lighting conditions (indicated as L1, L2, and L3 and described in the “Environmental Lighting Conditions” section to follow and depicted in Fig. 1). Cohort 1 will experience L1 followed by the L2 condition and cohort 2 will experience L2 followed by the L1 condition. Both cohorts will then experience the L3 condition. The primary outcome of this study is to determine how each lighting condition affects measures of sleep quality as assessed using wearable devices and self-report surveys. The secondary outcomes of this study are to determine how each lighting condition affects physical activity, cognitive function, social engagement, and mood as assessed using wearable devices, cognitive tasks, and self-report surveys. We will compare each of these measures between the L1 and L2 condition to test our central hypothesis that exposure to dynamic lighting (L2) will result in improvements of each measure compared to static lighting (L1). We will then compare these measures between L2 and L3 conditions to test our secondary hypothesis that improvements in measures of brain health saturate based on the amount of equivalent melanopic lux (EML) exposure.

Before starting the first lighting condition (L1 or L2 depending on the cohort), a baseline period lasting approximately 2 weeks will be used to characterize the lighting quality of each participants residence. The lighting in the baseline period will be set to match as closely as possible the lighting that was previously installed. Throughout the baseline, participants will be instructed to use the light as they normally would.

The L1 lighting condition and the baseline condition will have the same lighting parameters and will be configured to match the residents existing lighting prior to installing the current lighting system. Participants will be instructed to keep the lights all the time while awake during the duration of the study, however we gave individuals flexibility to wake up when they naturally did to not disrupt their sleep patterns by shifting to a rigid wakeup schedule.

After each 4-week lighting condition of the cross-over design, participants will experience a 1-week “washout” period during which they will experience the same lighting as the baseline period (L1 condition). After completing the cross-over and subsequent washout period, participants will experience the L3 condition to determine if there is a saturation effect of how much morning blue-rich light is needed to affect the health-related measures of interest.

### Measures of brain health using wearable devices

The goal of this study is to assess how lighting affects different measures of brain health. Although we are not performing any neuroimaging to directly measure brain activity, we are able to use different wearable devices, cognitive assessments, and surveys to infer how measures of brain health change, including changes in participants cognitive, mental, and physical health. During each day of the study, physiological measures will be recorded using wearable devices. Heart rate, steps taken, and sleep quality will be measured using a wrist-worn smartwatch (Withings ScanWatch, Withings, France SA). Participants will be asked to wear the smartwatch when awake and asleep, except when needing to bathe, wash, or swim, or when they need to charge the device. Measurements that will be analyzed from the watch will include participants’ steps taken and heart rate throughout each day of the study. Under-the-mattress devices (Withings Sleep Tracking Mat) will also be used to monitor each participant’s sleep. In combination with the smartwatch, sleep measurements that will be analyzed will include participants’ total sleep time, wake after sleep onset, and sleep stages (deep sleep, light sleep, and probable REM sleep as reported by the device). Data from these devices will be synchronized using the Withings Health Mate App (Withings, France SA).

Participants will also wear a LYS light sensor (LYS Technologies LTD, Copenhagen, Denmark) to monitor the amount of light to which they are exposed each day. Each participant will wear the LYS sensor on their collar to best capture their personal light exposure as close to their eye level as possible. This device will capture their light exposure both within and outside of their residence. The LYS button will measure EML, illuminance (lux), and correlated color temperature (CCT) which will be compared across each day of the study. To ensure compliance that participants wear the device, we can monitor the recorded values remotely each day. Should the signals recorded have very low variance, we will use this as an indicator to have a member of the study team reach out to the participant to ensure they are wearing the device.

These devices are commercially available and designed with safety in mind. Participants will be shown how to use each of the devices before the start of the study and will be encouraged to submit any questions to the study coordinator throughout the study.

### Measures of cognitive performance

The goal of analyzing cognitive performance using these tablet-based assessments is to assess how cognitive performance changes in a short time frame (i.e., how cognitive performance changes in each 4-week lighting condition). Due to the time constraints of this study design, we are unable to accurately assess changes in longer-term cognitive performance (e.g., cognitive slowing), however findings from this study could serve as preliminary indicators upon which a longer duration study could be performed. We will use tablet-based cognitive assessments to measure key aspects of executive functioning including working memory, response inhibition, and task switching. These assessments were designed around existing, validated cognitive assessments and were created in a custom-built application designed in the Well Living Lab.

Attentional control will also be assessed using a reaction time task. To capture individual differences in working memory, participants will perform the Operation SPAN task in which sequences of letters are remembered while also performing a distractor task comprised of mental math problems [77]. For the response inhibition dimension of executive functioning, participants will perform a classic three-color Stroop task in which participants indicate the font color of a color word while ignoring the word meaning (e.g., the word “red” in green font) [78]. To capture the task switching dimension, participants will complete a magnitude/parity task in which participants complete two different tasks based on the color of a number presented. If the number is yellow, participants indicate whether or not the number is larger than 5, and if the number is purple, participates indicate whether the number is even or odd. For the reaction time task, participants will perform the psychomotor vigilance task (PVT), which is a sustained-attention, reaction-timed task that measures the speed with which subjects respond to a visual stimulus [79].

Shortened versions of these tasks will be administered electronically on an iPad provided to the participants. Each task is expected to take approximately 5–10 min to complete, including a 1-minute training block that will be completed prior to each task throughout the study. Performance of these tasks will be assessed via accuracy, response latency, and overall duration to complete each task. These metrics will be compared across the different lighting conditions to understand how cognitive performance changes during the study.

Participants will be provided a schedule of when these assessments will be delivered throughout each week of the study and will be asked to perform these assessments in the morning (between 8am-12pm). Participants will be asked to perform assessments 2 times per week. Each participant will be able to access a customized webpage where they can check in to see which assessments they need to complete each day. Prior to study launch, a study team member will provide in-person instruction for completing these assessments. Compliance will be monitored remotely by the study team and participants will be reminded if they fail to complete multiple assessments.

### Self-report measures (surveys and questionnaires)

Participants will be asked to complete surveys and questionnaires to obtain self-report measures of sleep quality, social engagement, cognitive function, depression, and mood throughout the study. Surveys will be deployed electronically using Qualtrics and will include sleep questionnaires (Groningen sleep survey, Berlin Questionnaire, Stanford Sleepiness Scale (SSS) [80], and/or the Morningness Eveningness Questionnaire) [81], social engagement questionnaires (Lubben Social Network Scale [82], UCLA Loneliness Scale [83]). Participants will also complete mood- or depression-related questionnaires including the Patient health Questionnaire (PHQ8) [84], the Depression Anxiety and Stress Scale (DASS-21) [85] the Scale of Positive and Negative Affect (SPANE) [86], independence surveys (Lawton-Brody IADL Scale) [87], and the Ten Item Personality Inventory (TIPI) [88].

Similar to the cognitive assessments, surveys will be displayed on the same customized webpage and monitored by members of the study team to ensure compliance. A one-time baseline survey will be administered at study onset which will capture characteristics that are not expected to change over the course of the study (see Table 1 for the full survey schedule). Next, a brief (approximately 2–3 min) daily survey will capture changes over the previous 24 h. A weekly survey will be administered to capture other processes that may change over the course of a week (e.g., PHQ8, UCLA loneliness scale). Another survey will be administered at the end of each condition to characterize changes that might occur more slowly during each condition (e.g., Lubben Social Network Scale). Participants will also be asked to fill out a brief qualitative survey about each lighting condition they experience (e.g., “End of Condition” Survey). To ensure participant compliance for completing the surveys, we are able to monitor their survey performance remotely using a custom-built dashboard. By ensuring that each survey is completed (such as within 24 h of when they are delivered), we can reach out to participants who might forget to complete the assessments (see Data Auditing and Compliance section below).

**Table 1:** The list of surveys administered and when they are administered to each participant during the study

Survey	When Administered
Eligibility/Screening Survey	Before consent
Medical History	After consent
Socioeconomic survey	After consent
Morningness Eveningness Questionnaire (MEQ)	Start of Baseline
Berlin Questionnaire	Start of Baseline
TIPI Big 5 Personality	Start of Baseline
Stanford Sleep Scale	Daily
SPANE	Daily
Groningen Last Night Sleep Report	Daily
DASS21	Weekly
PHQ-8	Weekly
UCLA Loneliness Scale	Weekly
Falls / Hospitalization Survey	Weekly
Lawton Brody Instrumental Activities of Daily Living Scale	End of Condition
Lubben Social Network Scale - Revised (LSNS-R)	End of Condition
End of Condition Survey	End of Condition
End of Study Opinion Survey	End of Study

### Data auditing and compliance

All incoming data streams will be continuously monitored by the research team for missingness using cloud-based APIs and real-time database visualization queries. Participants who possess devices with more than 48 h of missing data will be contacted for troubleshooting, and study representatives will support participants as needed. Participants missing 3 out of 4 days or 3 consecutive daily surveys will be contacted with offers to support. Repeated failures to complete study tasks will involve the study’s primary investigator (PI) reaching out to participants to develop a personalized strategy to increase compliance.

### Mental health mitigation plan

Some of the surveys being proposed to use in the study (such as the PHQ8) can be used as a diagnostic tool and as such require a mental health risk mitigation plan. The same research coordinator responsible for cohort allocation will monitor the scores from this survey each time it is administered for each participant (weekly). At the start of the study, contact information will be collected for each participant’s primary care physician (PCP). If a participant scores as having severe depression on the inventory (PHQ score > = 20), the research coordinator will notify the PI (Dr. Chen) who will then inform the participant and their PCP of the reported severe depression levels. If the depressive episode has not been previously diagnosed or treated, the PCP will be asked to follow up with the participant to discuss changes in their mood and create a response plan. Participants will subsequently be asked whether they would like to continue with the study. If they are interested in continuing, the participant will again complete the UBACC consent capacity protocol (previously discussed) to ensure that they are able to consent. This procedure will also ensure that the participant fully understands their option to leave the study at any time.

### Measures of clinical events

The intervention proposed here is non-intrusive and not anticipated to elicit any adverse events. Participant adverse events may include adverse reactions to the lighting conditions in the room, falls, hospitalizations, etc. All adverse events that occur during this study will be recorded and documented. Because this is an exploratory study, the full extent of adverse events is unknown and all events occurring during the duration of the study will be recorded and analyzed. Adverse events could typically happen with the older participant population independent of this study. We will electronically send a form to each participant every 2 weeks throughout the study to ascertain if any adverse events, such as a fall or hospitalization, has occurred. Comparing the incidence of adverse events documented during the study with historical adverse events incidence rates (such as those reported in the Mayo Clinic Olmsted Study of Aging [89] will help us identify clinical benefits to the proposed lighting conditions. If participants identify any adverse event related to the lighting conditions, wearable devices, or other study procedures, the research team will contact the participant and clearly re-iterate that study participation is fully voluntary and that participants can leave the study at any time for any reason.

### Environmental lighting conditions

Numerous studies have concentrated on the physiological and psychological effects of light intensity and Correlated Color Temperature (CCT, measuring how warm or cool the light appears) in older adults [23, 35, 36, 62–75]. CCT characterizes the color of light, which is based on the wavelength at which the light travels. Light that has shorter wavelengths (e.g., 450 to 530 nm) would be perceived as blue, whereas light that has longer wavelengths (e.g., 620 to 750 nm) would be perceived as red [28]. Experiencing more blue light in morning has been shown to have positive physiological and psychological effects on one’s health. Although light exists on a continuum, for the purposes of this research protocol we are defining blue light as light with wavelengths between 450 and 530 nm.

In this study, participants will experience three different lighting conditions in the kitchen and dining areas of their residences to assess how each condition affects different measures of brain health. Light bulbs originally installed in the participant’s living and kitchen areas (which emit static lighting) will be replaced by Philips Hue dynamic light bulbs (E26, warm-to-cool white, 16 W; Signify N. V.; Eindhoven, Netherlands), which can output 0 lumens to 1600 lumens (a measure of brightness of the light) and CCT from 2200 to 6500 K. Additional lamps will be installed in the kitchen and dining areas of the participants’ residences to achieve the desired brightness and color temperatures based on simulations described below. All lights used in this study are commercially available and will be installed by members of the study team.

**Fig. 2:**
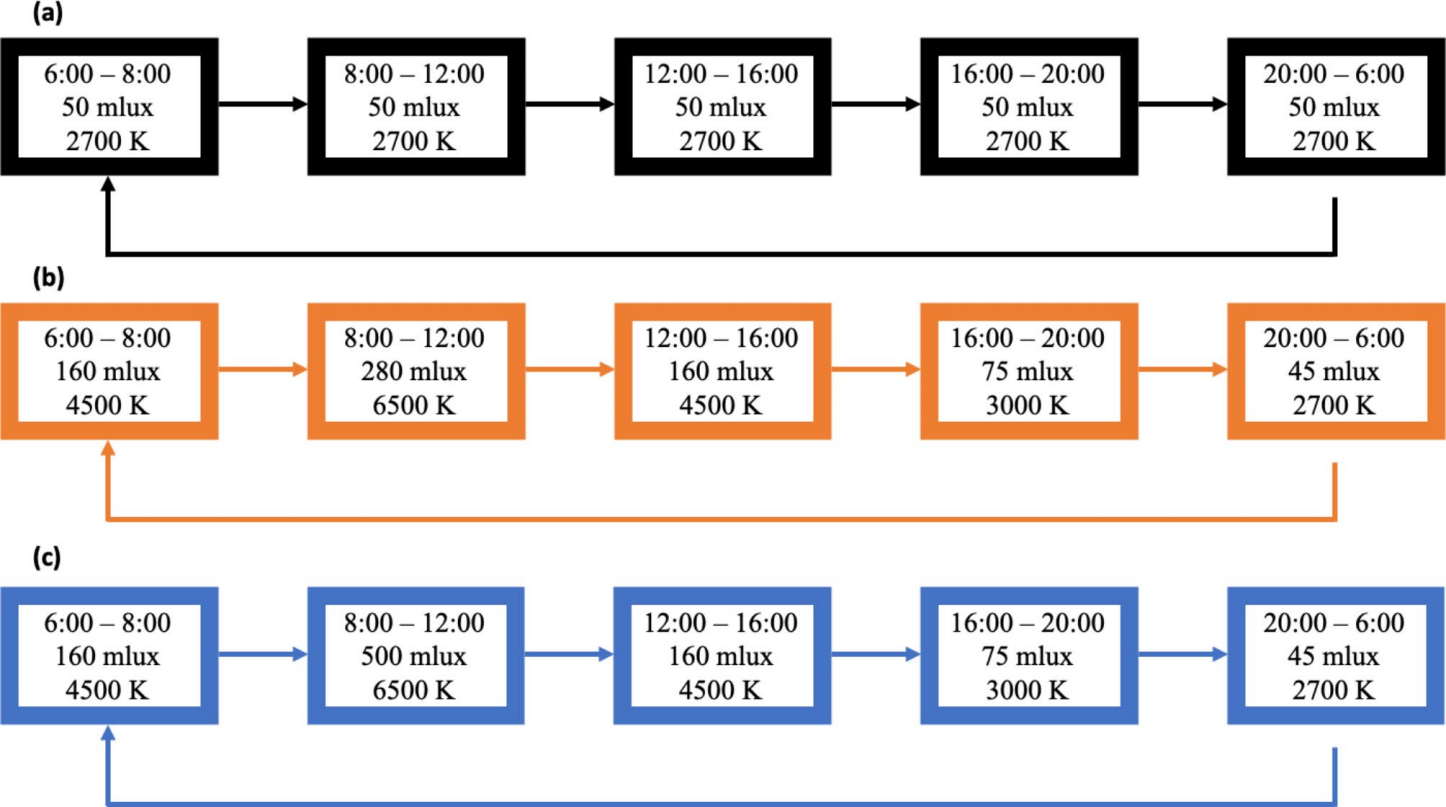
24-hour schedule of lighting parameters for **a**) L1, **b**) L2, and **c**) L3 lighting conditions. L1 is designed to be a static light condition similar to what residents would typically have in their independent living residences. L2 and L3 are designed to have increased EML in the morning hours with more exposure to blue-rich light and also slightly decreased EML in the evening hours

Each lighting condition will be programmed to achieve minimum CCT and brightness levels throughout a 24-hour period (Fig. 2). The three lighting conditions that participants will experience are:

1. L1: static lighting condition with fixed CCT and brightness;
2. L2: dynamic lighting condition with dynamic CCT and brightness; and.
3. L3: dynamic lighting condition similar to L2 but with increased brightness in the morning hours.

The light bulbs installed in each residence will be programmed and controlled using the Philips Hue Application Programming Interface (API). Each lighting condition will be implemented to achieve minimum levels of equivalent melanopic lux (EML, measured in mlux) while ensuring high levels of blue-rich light in the morning and low levels of blue-rich light in the evening. EML quantifies the circadian effect from the light source by weighting the response from ipRGCs [90, 91]. Recent studies have suggested EML is a favorable indicator of the melatonin suppression response [37, 38, 92]. A minimum EML of 280 melanopic lux (mlux) for at least 4 h during the daytime is recommended as an optimal circadian stimulus that normalizes biological responses such as melatonin suppression, circadian phase resetting, and subjective altering responses [39, 93]. As shown in Fig. 2, the L1 condition is designed to have a fixed EML throughout the 24-hour period, whereas the L2 and L3 conditions provide a cycle of gradually higher EML during the morning and lower EML in the evening. As such, the mlux values in the morning are greater in L2 and L3 compared to L1 (and less in the evening for L2 and L3 compared to L1). Guidelines provided by the WELL Building Standard were taken into consideration when selecting the EML levels for each lighting condition [93].

The purpose of the L3 condition is to increase the amount of morning EML and decrease the amount of evening EML to determine if there is a saturation effect with regards to the different measures of brain health, we are collecting in L1 and L2. Exposure to EML beyond 280 mlux may not result in any added health benefits [39]. However, older adults tend to perceive light differently than younger adults and require brighter lighting [43–47]. As such, having each participant experience the L3 condition will give us an opportunity to examine whether light intensity plays a factor in any detected changes of brain-health measures.

### Verifying lighting conditions through simulations and field measurements

**Fig. 3:**
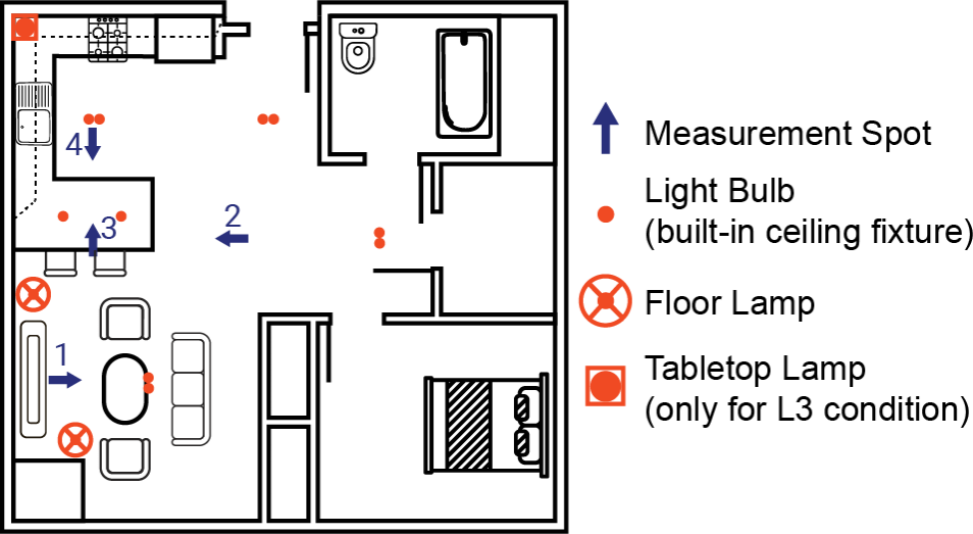
Drawing of an example floorplan where the lighting intervention will be implemented. Measurements are taken in four directions at specific locations in the room. Example locations and directions are shown in blue. Each floor plan has built-in lighting fixtures in the ceiling as indicated by the orange circles. Additional floor lamps will be added to the room to increase the amount of light to which participants are exposed. For the L3 condition, the amount of blue light needs to be increased compared to the L2 condition. This will be accomplished by placing a tabletop lamp in the corner above the cabinets in the kitchen. Only the kitchen and dining areas will have the lighting system installed for this study, as these are where we anticipate participants will spend most of their time

To ensure the target EML and CCT values in Fig. 2 were achievable with our proposed lighting design, we conducted computer simulations and collected lighting measurements from one empty residence in the facility. Figure 3 depicts one floorplan where we identified locations where we anticipated participants will spend much of their time: (1) center of the sofa facing the living room, (2) middle of the hallway entrance facing the living room, (3) center of the pendent lights above the kitchen island facing the kitchen, and (4) underneath the light fixture in the kitchen facing the living room. We implemented our lighting intervention in this empty room while measuring the illuminance and CCT using a research grade spectrometer (CL-500 A; Konica Minolta, Inc; Tokyo, Japan) placed 1.2 m above the floor from each of these target locations to approximate the individuals seated eye-level. We set the lights to the morning scene (8:00am-12:00pm) from the L2 condition because ensuring we could achieve these target EML and CCT values confirmed we could also achieve the lower EML and CCT values as well. Using these measurements from the spectrometer, we were able to confirm that we could achieve the desired brightness levels using a 3D simulation model. This model simulated the EML values for the entire kitchen, living area, and hallway using lighting simulation software Radiance and DIVA for Rhino with proven validity. Because each residence will be slightly different in floorplan, we made certain assumptions regarding surface reflectance, melanopic ratio, and lumen adjustments for the purposes of this simulation.

**Fig. 4:**
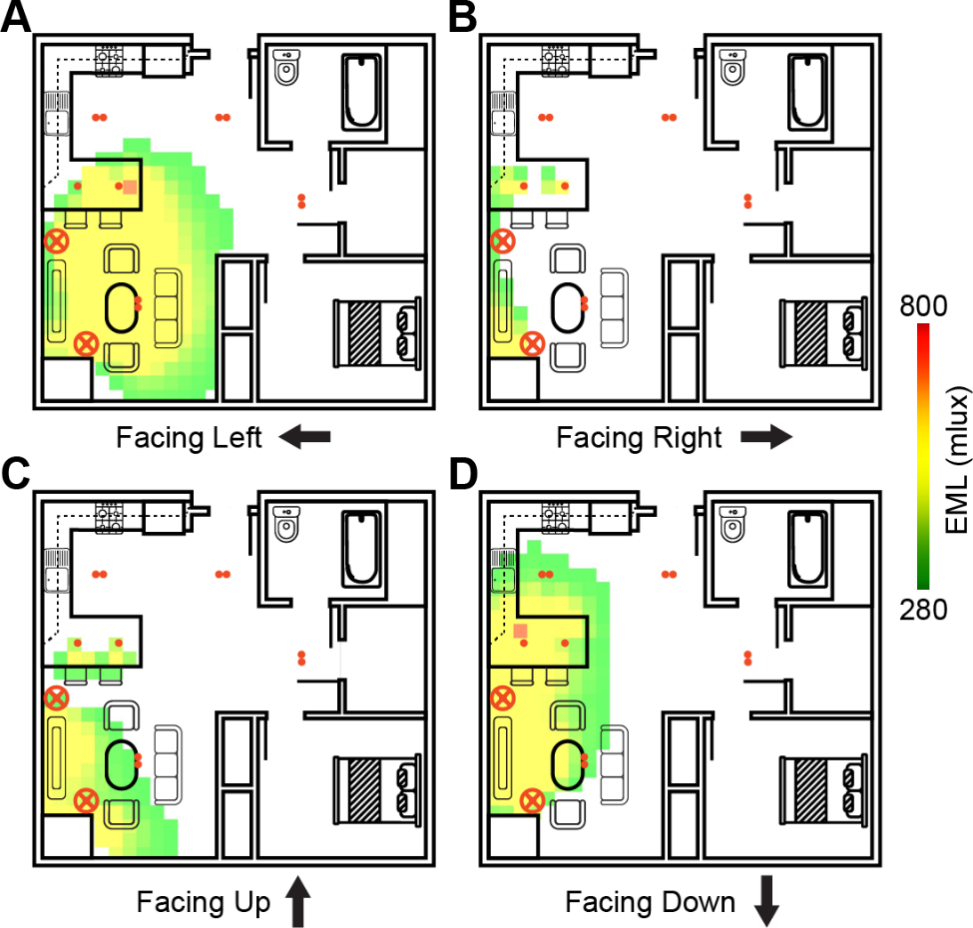
Results from the 3D simulation of the EML values in four different directions. The lights were simulated at the L2 morning condition (6500 K, 280mlux) to determine where in the room participants would experience greater than 280mlux. Heatmaps overlaying the floorplan from Fig. 3 indicate that the sitting area in both the kitchen and dining area will have sufficient light to reach our target brightness levels

We used this simulation approach to ensure that we could reach our minimum brightness levels. As shown in Fig. 4, we simulated the morning light scene from the L2 condition (6500 K, 280mlux) and the lighting system exceeded our target EML value of 280 mlux in the kitchen and dining areas overlapping where we anticipate a participant sitting. To achieve these EML values, we needed to include two floor lamps with 5-in-1 splitters to increase the amount of light emitted from each lamp (e.g., output from five Philips Hue dynamic light bulbs instead of one).

For the L3 condition, the morning EML values (500mlux) are increased even further to determine if there is a saturation effect of how much light exposure one needs to also achieve any health-related benefits. The tabletop lamp was not included in Fig. 4 because it was not a part of the L2 condition, however, we were able to use simulations to determine how much additional light would be needed. Based on these simulations, an additional modified tabletop lamp placed in the corner of the kitchen with a light bulb capable of emitting 12,000 lumens at 6500 K would reach the target EML level. Overall, these simulations confirmed we will have sufficient lighting with the designed lighting configuration to achieve our target EML and CCT levels for each lighting condition.

For each room in the main study, we will continue to take measurements using the research grade spectrometer (CL-500 A; Konica Minolta, Inc; Tokyo, Japan) as well as using Blue Iris Fixed Sensors (Blue Iris Lab, California, US) to monitor how ambient light changes each day and across each room. The Blue Iris sensors allow for measuring EML and CCT continuously and will be placed strategically in each room, so we are able to characterize how different floorplans or changes in daylight affect the lighting conditions throughout the study. Additionally, participants will wear the LYS sensor (LYS Technologies LTD™; Copenhagen, Denmark) throughout the time they are awake and moving in the room to capture how much light they are exposed to throughout the study. Both the Blue Iris and LYS sensors will be calibrated in the lab using regression methods similar to those described by Bäumker et al. prior to the start of the study [94]. Although there are different types of floorplans in the facilities where this study will be conducted, the electrical configurations are expected to be similar among all the residences. Using both the wearable and static light sensors and simulating as described, we expect to be able to successfully determine how exposure to different indoor lighting conditions affects the different measures of brain health.

### Statistical analysis plan

Using data from both wearable devices and surveys, the primary comparison will be within-person change in sleep quality scores between each of the three lighting conditions (L1, L2, L3) and the same scores measured during the baseline period. The L1 condition serves as an active control condition as this is the same lighting as they would typically experience in the baseline condition. The secondary comparison will be to compare changes in physical activity, heart rate, and survey data across each lighting condition pair. Data collected during the baseline period will also be compared against the L1 condition to determine whether asking participants to change their behavior with the lights (e.g., do not turn the lights off) affects any of the dependent variables of interest, thereby ensuring that any health benefits seen in the dynamic conditions relative to the static condition are likely reflective of improvements over participants lighting as usual. Participants who are eligible only because their partner is eligible will be analyzed separately.

We will use generalized estimating equations to account for the longitudinal repeated-measures design and handle missing data. For the surveys and cognitive assessments, there will be a minimum of 8 measurements per lighting condition (two cognitive assessments per week for four weeks). If only 35 participants are enrolled, we expect 80% power to detect a difference of minimum of 0.45 standard deviations. Conversely, if we have 70 participants enrolled (i.e., all the rooms are double occupancy with both persons eligible) we expect 80% power to detect a minimum difference of 0.3 standard deviations. For either of these scenarios, we expect to be able to detect at least 0.3 standard deviations, yielding sufficient power, even for the relatively infrequent cognitive assessments. To minimize missing data,, we will monitor key metrics (such as response rates) throughout the study using IRB-approved approaches to encourage participation. As a rule of thumb, participants who complete the study with greater than 50% missing data on any outcome measure will not be included in the relevant final analysis population.

### Data management and confidentiality

All data captured will be stored in Mayo Clinic’s secure databases. Data from environmental sensors and/or wearable devices will be stored in and collected from the Mayo Clinic’s instance of Microsoft Azure Cloud. The Azure Cloud provides secure cloud-based storage. The data should not contain any participant identifying information. All participants will be assigned a unique identifier simply indicating which cohort they are in and the participant number (e.g., 2001 would be participant 1 in cohort 2).

De-identified survey responses will be stored in a secure database (with restricted access limited to members of this study team) once the participant has finished their period of active participation during relocation. Survey and questionnaire responses will be coded and de-identified upon collection to protect the participant’s privacy. Any data that contains any personal health information will be stored in one of our HIPAA compliant secure databases and maintained by our independent research coordinator. The Well Living Lab databases residing on Mayo Clinic’s Azure Cloud are HIPAA compliant and secured by passwords and user restrictions. Again, all participant information will be de-identified, and the identifying key will not be shared with collaborators. All participant data collected during this study, including that gathered through surveys, questionnaires, interviews, and physiological and environmental sensors, will be stored indefinitely, and may be used in future studies. There are no plans to share this study data with outside collaborators, and any sharing plans will be discussed with the IRB prior to sharing.

## Discussion

We are presenting a novel research protocol to assess how indoor lighting affects several measures of brain health in older adults living in independent living residences. Preservation of brain health is an important component in maintaining function and independence. Many studies of the health benefits of different lighting conditions have focused on individuals who have already developed dementia in assisted living facilities. Our focus here is to understand how similar lighting interventions might be effective at promoting different measures of brain health which have been shown to help mitigate health-risks before the onset of dementia may occur [24]. Understanding how indoor lighting might affect or even improve multiple measures of brain health, such as sleep, physical activity, social engagement, and mood could have significant implications for guiding older adults to maintain their health and well-being as they age.

The design of this study also provides an innovative approach to providing a control to compare against for each condition. The L1 (static lighting) condition will be designed to match as closely as possible the lights that participants initially had installed. However, participants will need to change their behavior during each of the lighting conditions (L1, L2, L3), making the L1 condition an active control condition. Activity recorded during the baseline period during which participants can turn on/off their lights as needed will also be compared with activity recorded during each of the lighting conditions to determine how much of an effect exists during the active control (L1) condition.

There are limitations and challenges that need to be addressed as part of this study. First, the goal of this study is to determine how several measures of brain health change in response to different indoor lighting conditions. However, due to time constraints of the study, we are only able to assess the effect of each lighting condition over a 4-week period. This will allow us to capture short-term changes and demonstrate the viability of how lighting could improve measures of brain health, but a follow up study of a much longer duration is needed to accurately characterize effects of lighting on more chronic changes (such as cognitive slowing). Second, due to the operational logistics, only one senior living facility in Rochester, MN will be considered for this study. As such, the demographics and potential co-morbidities of the residents of the facility may not match those of the greater population. However, key insights were be identified which can then be compared against findings from regional studies such as the Mayo Clinic Olmsted Study of Aging [89] to determine how consistent or different the findings are. Second, recruitment may be difficult if not enough residents who are currently living in the facility are interested in participating in the study, in which we might need to consider expanding our locations. Third, when analyzing sleep measures, we are unable to perform clinical polysomnography (PSG) remotely. Therefore, we are using several different measures of sleep to triangulate different sleep patterns. Although these or similar devices we have selected have been validated in prior studies performed by other researchers [95, 96], we will consider these potential limitations when analyzing the sleep findings. Finally, there may be some challenges with ensuring compliance by participants in completing the various tasks and adhering to wearing the wearable devices. However, we plan to have several information sessions and to provide reminders to the participants especially early in the study to ensure they are completing the tasks as outlined above. Overall, we expect to be able to handle these limitations and challenges to successfully complete this research protocol. The benefits of this study greatly outweigh the risks, as these research findings could generalize to guide how indoor lighting can improve the health and well-being for the greater population and provide vital information on how to design living spaces that promote healthy aging.

### Trial status

This trial began recruitment in April 2023. Recruitment will cease when the expected number of participants is reached. Our estimated target date for completed recruitment is December 2023.

## Data Availability

Data related to the lighting simulations is available upon request to the corresponding author (linhao.li@delos.com). No other data were generated or analyzed related to this study protocol article. Deidentified data and materials for the study proper will be made available upon request.

## Abbreviations

API: Application programming interface
CCT: Correlated color temperature
EML: Equivalent melanopic lux
IRB: Institutional review board
ipRGCs: Intrinsically photosensitive retinal ganglion cells
L1, L2, L3: Lighting conditions 1, 2, and 3
mlux: Melanopic lux
OSPAN: Operational span task
PVT: Psychomotor vigilance task
RGCs: Retinal ganglion cells
UBACC: University of California, San Diego Brief Assessment of Capacity to Consent
WHO: World Health Organization
